# Uterine papillary serous and clear cell carcinomas predict for poorer survival compared to grade 3 endometrioid corpus cancers

**DOI:** 10.1038/sj.bjc.6603012

**Published:** 2006-02-21

**Authors:** C A Hamilton, M K Cheung, K Osann, L Chen, N N Teng, T A Longacre, M A Powell, M R Hendrickson, D S Kapp, J K Chan

**Affiliations:** 1 Division of Gynecologic Oncology, Department of Obstetrics and Gynecology, 875 Blake Wilbur Drive, MC 5827, Stanford, CA 94305, USA; 2 Stanford Cancer Center, 875 Blake Wilbur Drive, MC 5827, Stanford, CA 94305, USA; 3 Division of Gynecologic Oncology, Department of Obstetrics and Gynecology, University of California, San Francisco Comprehensive Cancer Center, 1600 Divisidero, San Francisco, CA 94115, USA; 4 Division of Hematology/Oncology, Department of Medicine, Chao Family Comprehensive Cancer Center, University of California, Irvine – Medical Center, 101 The City Drive, Orange, CA 92868, USA; 5 Department of Pathology, 300 Pasteur Drive, Stanford, CA 94305, USA; 6 Divison of Gynecologic Oncology, Department of Obstetrics and Gynecology, Washington University School of Medicine, 4911 Barnes Hospital Plaza, St Louis, MI 63110, USA; 7 Department of Radiation Oncology, 875 Blake Wilbur Drive, MC 5827, Stanford, CA 94305, USA

**Keywords:** uterine, papillary serous, clear cell, survival

## Abstract

To compare the survival of women with uterine papillary serous carcinoma (UPSC) and clear cell carcinoma (CC) to those with grade 3 endometrioid uterine carcinoma (G3EC). Demographic, pathologic, treatment, and survival information were obtained from the Surveillance, Epidemiology, and End Results Program from 1988 to 2001. Data were analysed using Kaplan–Meier and Cox proportional hazards regression methods. Of 4180 women, 1473 had UPSC, 391 had CC, and 2316 had G3EC cancers. Uterine papillary serous carcinoma and CC patients were older (median age: 70 years and 68 *vs* 66 years, respectively; *P*<0.0001) and more likely to be black compared to G3EC (15 and 12% *vs* 7%; *P*<0.0001). A higher proportion of UPSC and CC patients had stage III–IV disease compared to G3EC patients (52 and 36% *vs* 29%; *P*<0.0001). Uterine papillary serous carcinoma, CC and G3EC patients represent 10, 3, and 15% of endometrial cancers but account for 39, 8, and 27% of cancer deaths, respectively. The 5-year disease-specific survivals for women with UPSC, CC and G3EC were 55, 68, and 77%, respectively (*P*<0.0001). The survival differences between UPSC, CC and G3EC persist after controlling for stage I–II (74, 82, and 86%; *P*<0.0001) and stage III–IV disease (33, 40, and 54; *P*<0.0001). On multivariate analysis, more favourable histology (G3EC), younger age, and earlier stage were independent predictors of improved survival. Women with UPSC and CC of the uterus have a significantly poorer prognosis compared to those with G3EC. These findings should be considered in the counselling, treating and designing of future trials for these high-risk patients.

Endometrial cancer is the most common gynecologic cancer in the US. In total, 40 880 new cases are projected in 2005, with 7310 women dying from this disease (Jemal *et al*, 2005). The majority of corpus cancers are early-stage, low-grade, endometrioid tumours with a good prognosis. On the other hand, uterine papillary serous carcinoma (UPSC), clear cell carcinomas (CC), and grade 3 endometrioid carcinoma (G3EC) have been identified as high-risk endometrial cancers and account for the majority of uterine cancer deaths. Uterine papillary serous carcinoma and CC histologies have also been identified as distinct variants of endometrial cancer with a propensity for extrauterine spread and poor prognosis (Clement and Young, 2004). Furthermore, previous studies have shown that women with G3EC have a significant risk for nodal metastases at 28% (Creasman *et al*, 1987). However, other authors have not been able to show a survival difference between UPSC and CC compared to G3EC (Alektiar *et al*, 2002; Creasman *et al*, 2004).

Over the past decade, some clinical trials have incorporated UPSC, and CC while others have excluded these poor histologic subtypes (Keys *et al*, 2004; Randell *et al*, 2006). This dichotomy stems from the conflicting data in the current literature regarding prognosis of high-risk endometrial tumours. Of the limited published reports, most are based on small retrospective series from single institutions that lack power to detect significant differences. In this large population-based study, we report on the outcomes of 4180 patients with UPSC, CC, and G3EC to determine if there are significant prognostic differences between these histologic cell types, and, if so, what clinico-pathologic prognostic factors are responsible.

## MATERIALS AND METHODS

After Institutional Review Board approval, 4180 women with high-risk (UPSC, CC, and G3EC) endometrial cancers were extracted from the Surveillance, Epidemiology and End Results (SEER) database between 1988 and 2001. Additionally, 11 014 patients with grade 1 or 2 endometrioid (G1EC, G2EC) tumours were analysed for demographic context. Data from the SEER database are reported from twelve population-based registries that represent approximately 14% of the US population: San Francisco-Oakland, Connecticut, metropolitan Detroit, Hawaii, Iowa, New Mexico, Seattle (Puget Sound), Utah, metropolitan Atlanta, Alaska, San Jose-Monterey, and Los Angeles (Hankey *et al*, 1999).

Information including age at diagnosis, race, stage of disease, histology, and adjuvant therapy were extracted and analysed. Race was categorised as White, Black, Asian (Chinese, Japanese, Korean, Vietnamese, or Filipina), or other race. Adjuvant radiotherapy was categorised as either receiving or not receiving adjuvant radiotherapy.

To analyse trends in the study cohort and to determine 5-year disease-specific survival, *χ*^2^ tests and Kaplan–Meier analyses were used to assess differences between UPSC *vs* CC *vs* G3EC. *P*-values <0.05 were considered statistically significant, indicating statistically significant differences between the three histologic cell types. The outcome of interest was death from endometrial cancer and time to death was censored in women who died from causes other than uterine cancer. The Cox proportional hazards model was used to assess the significance of multiple variables simultaneously. All data were analysed using Intercooled Stata (Version 8.0; Stata Corporation, College Station, TX, USA) and SAS (Version 6.12; SAS, Inc., Cary, NC, USA).

## RESULTS

From 1988 to 2001, 4180 uterine cancer patients had high-risk histologic cell types including 1473 with UPSC, 391 with CC, and 2316 with G3EC. The demographics of the study population are presented in Table 1. The median age at diagnosis of the UPSC and CC patients was significantly higher compared to those with G3EC (70 years and 68 *vs* 66 years, respectively; *P*<0.0001). Blacks comprised a significantly higher proportion of patients with UPSC (15%) and CC (12%) compared to G3EC (7%; *P*<0.0001).

All patients in this study underwent a hysterectomy and surgical staging procedure. Of the patients with stage I–II disease with UPSC, CC, and G3EC, 55, 61 and 59% underwent lymph node assessment, respectively. Of these patients, the median number of nodes resected in those with UPSC, CC, and G3EC were 11, 11, and 13, respectively. Fifty-two percent of patients with UPSC had stage III–IV disease compared to only 36 and 29% of those with CC and G3EC, respectively (*P*<0.0001). Of women with UPSC, CC, and G3EC, 39, 48, and 47% underwent adjuvant radiotherapy (*P*<0.0001), respectively. However, we were unable to obtain information regarding types of radiation, fields, or dosages. Similarly, details on chemotherapy use or specific regimens were not available.

**Figure 1:**
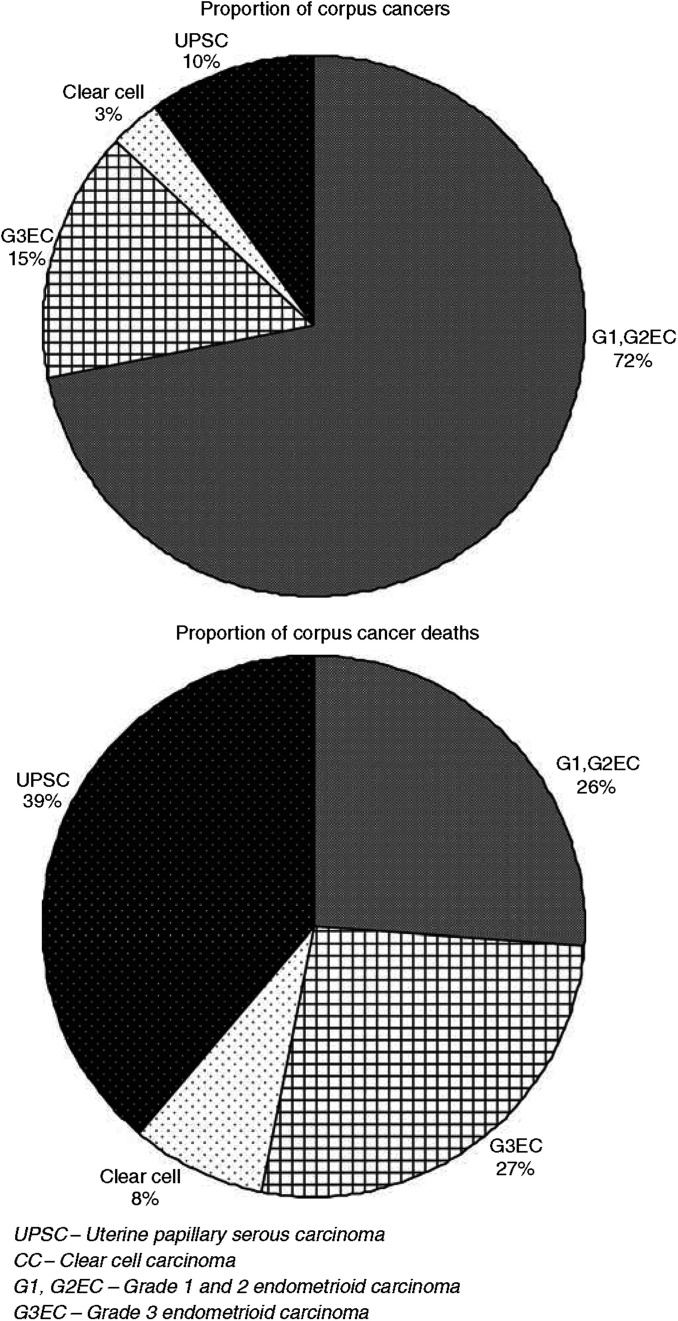
Proportion of corpus cancers compared to proportion of corpus cancer deaths by histologic cell type.

**Figure 2:**
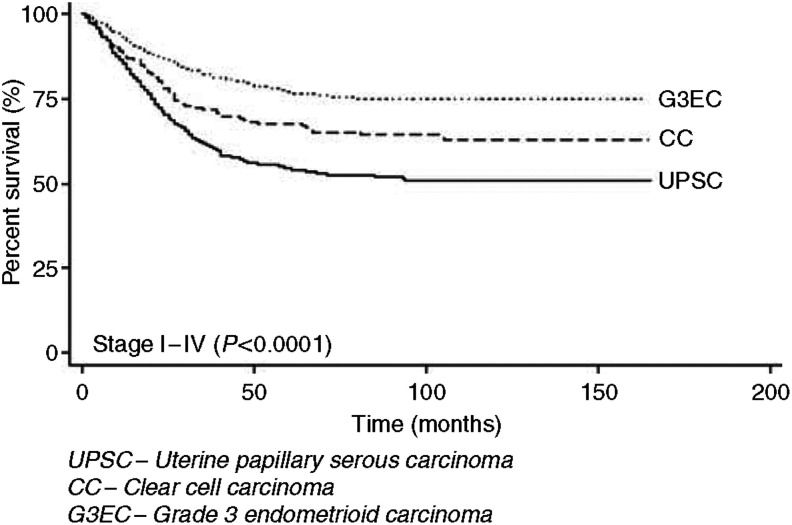
Kaplan–Meier disease-specific survival by histology.

**Figure 3:**
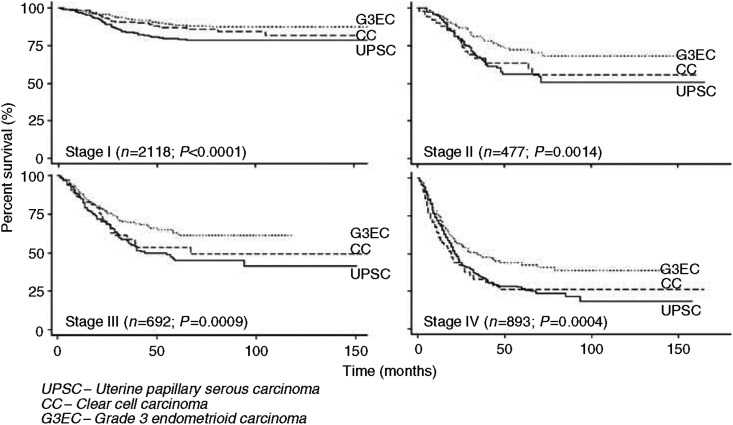
Kaplan–Meier disease-specific survival by histology and stage.

Although patients with UPSC, CC, and G3EC represented only 10, 3, and 15% of endometrial cancers in our study population, they accounted for 39, 8, and 27% of cancer deaths, respectively (Figure 1). In the time interval studied, the percentage of patients dying from their respective histologic cell type of endometrial cancer (number of deaths for specific histology/number of patients diagnosed with specific histology) were 34, 28, and 15% for UPSC, CC, and G3EC, respectively. Patients with UPSC and CC had a significantly decreased 5-year disease-specific survival of 55 and 68% compared to 77% for G3EC (Figure 2). These survival trends remain significant even when stratified by stage (Figure 3). Patients with stage I–II UPSC, CC, and G3EC (*n*=2595) had survivals of 74, 82, and 86%, respectively, compared to 33, 40, and 53% in those with stage III–IV disease (*n*=1585) (*P*<0.0001 for stage I–II; *P*<0.0001 for stage III–IV). Of the 2118 women with stage I disease, UPSC patients had a significantly worse survival compared to CC and G3EC patients with survival rates at 80 *vs* 91% and 92% for stage IB (*P*=0.0001), and 66 *vs* 82% and 82% for stage IC disease (*P*=0.0017), respectively. However, we were unable to demonstrate a statistical difference in survival between UPSC, CC, and G3EC in stage IA disease (90, 87, and 94%; *P*=0.28).

Using a Cox proportional hazards model, demographic and clinico-pathologic prognostic factors were investigated as independent predictors of survival after adjusting for contributing factors such as age, race, stage, histology, and radiotherapy. On multivariate analysis, advanced stage disease (*P*<0.001), aggressive histologic cell types (UPSC and CC, *P*<0.001), and older age at diagnosis (*P*<0.001) were all independent predictors of poorer survival (Table 2). However, race (*P*=0.888) and radiotherapy (*P*=0.450) were not significant independent prognostic factors.

## DISCUSSION

In 1982, Hendrickson *et al* (1982) identified UPSC as a clinically aggressive and morphologically distinct variant of endometrial adenocarcinoma. Clear cell endometrial carcinoma was first described in the English literature in 1957. Both cell types have a predilection for distant spread and recurrence (Kay, 1957; Silverberg and De Giorgi, 1973; Kurman and Scully, 1976; Abeler *et al*, 1996). Over the last two decades, these histologic types have been grouped together with grade 3 endometrioid cancers as high-risk tumours. However, debate remains whether there is a significant difference in prognosis between these high-risk subtypes and more importantly, if these cell types should be treated as separate disease entities (Munkarah, 2004).

This is one of the largest series that compares the clinico-pathologic prognostic factors and outcomes of patients with UPSC and CC *vs* G3EC. Previous reports have shown that UPSC is an uncommon uterine cancer but accounts for a disproportionate number of endometrial cancer deaths (Hendrickson *et al*, 1982; Carcangiu and Chambers, 1995). Similarly, in our series, patients with UPSC comprised of only 10% of corpus cancers in our study but accounted for 39% of endometrial cancer deaths. When compared to women diagnosed with G3EC, patients with UPSC accounted for 5% less cases but 12% more deaths. The patients with UPSC and CC were older with median ages at 70 and 68 years compared to 66 years in patients with G3EC. Women with UPSC and/or CC have been described as type II corpus cancers that are associated with older age (Bokhman, 1983). Although prior reports have consistently demonstrated older age in patients with UPSC and CC compared to endometrioid uterine cancers, this current study is one of first series with sufficient numbers of patients to show a statistically significant increase in age between the high-risk histologies (Abeler and Kjorstad, 1991; Carcangiu and Chambers, 1995; Matthews *et al*, 1997).

In this current series, the percent of patients with disease spread beyond the uterus (stage II–IV) at the time of diagnosis was 64, 50, and 40% for UPSC, CC, and G3EC, respectively (*P*<0.001). These findings suggest that the poorer prognosis associated with these histologies may be due to advanced disease at the time of diagnosis. In a single institution report of 136 patients, Boruta *et al* (2004) also found that patients with UPSC were more likely than those with G3EC to present with extrauterine disease (OR=2.2; 95% CI=1.1–4.5). Additionally, Cirisano *et al*, also reported a higher likelihood of upstaging and finding gross metastases among UPSC and CC tumours compared to G3EC controlled for comprehensiveness of surgical staging (Cirisano *et al*, 1999, 2000). Similar to our series, these reports point towards the more aggressive behaviour of UPSC and CC compared to G3EC. In this current report, we showed that the poorer prognosis of UPSC and CC is not completely explained by the more advanced stage of disease at presentation because the worse prognosis of these cell types holds even after controlling for stage at presentation (Figure 3).

Other prognostic factors that may impact survival include complete surgical staging and extent of lymph node dissection (Creasman *et al*, 1987; Chan *et al*, 2003; Huh *et al*, 2003). All patients in this study underwent surgical staging based on FIGO criteria and the majority of patients with early stage disease had a lymph node assessment. Previous studies have demonstrated a survival benefit associated with a thorough lymphadenectomy, we proposed to determine if the extent of lymph node dissection may have contributed to the better survival of those with G3EC. Our data showed that the median number of nodes resected between UPSC, CC, and G3EC patients were not statistically different, but UPSC patients continued to have a poorer prognosis. Therefore, using node count as a surrogate, it does not appear that stage migration or an inequality in the extent of surgery contributed to the poorer prognosis of UPSC or CC. Most importantly, higher-risk histologies (UPSC and CC) remained as independent prognostic factors for poorer survival in multivariate analysis.

Previous studies have also investigated the prognostic difference between the high-risk histological types and found similar results. In a series of 139 patients with early stage disease, those with UPSC had a progression-free and overall survival that were significantly worse than those with G3EC (35 *vs* 82%, *P*=0.03 for progression-free and 43 *vs* 89%, *P*=0.02 for overall survival) (Boruta *et al*, 2004). Similarly, Cirisano *et al*, evaluated survival analyses in stage I–II patients and demonstrated a worse 5-year survival in those with UPSC and CC compared to endometrioid carcinoma (56 *vs* 86%; *P*=0.11). On the other hand, Alektiar *et al* (2002) did not find a survival difference between these histologic cell types; however, only 28% of patients in his series underwent a comprehensive surgical staging procedure. More recently, Creasman *et al*, performed a large population-based analysis of 523 stage I patients with high-risk cell types from the FIGO database. Although these authors did not find a survival difference in those with stage IA and IB disease, the survival of women with stage IC was poorer in those with UPSC compared with CC or G3EC (Creasman *et al*, 2004). In our series, we showed a worsened survival in those with stage IB and IC UPSC compared to G3EC. However, similar to Creasman's series, we were unable to demonstrate a statistically significant difference between those with stage IA disease. Table 3 summarises prior studies that have evaluated the 5-year survival of high-risk histologies.

As with other large population-based series, our report was limited by a lack of central pathology review. To determine if there are significant discrepancies between registry and referral pathologists, Piver *et al* (2000), reviewed slides from a large cancer registry and found only 1% of cases had major differences with regard to either site of origin or histopathologic type. Similarly, Tyler *et al* (1991) performed slide reviews on 477 women diagnosed with ovarian cancer and compared the diagnoses of pathologists contributing to tumour registries affiliated with the SEER program to an expert panel of three gynecologic pathologists and found and overall agreement of 97%.

Another shortcoming of this study is the lack of information regarding adjuvant chemotherapy. Nevertheless, even in smaller single institution reports, the treatment of patients with UPSC and CC differed significantly. Recently, McMeekin *et al* (2005) performed an analysis of 1203 patients diagnosed with advanced or recurrent UPSC, CC, and endometrioid uterine cancers from the Gynecologic Oncology Group, and found that the responses to chemotherapy between these histologic cell types did not differ. However, histology remained as an important prognostic factor. More specifically, the relative hazard ratio for UPSC and CC was 1.20 (1.02–1.40; *P*=0.03) and 1.51 (1.1–2.1; *P*=0.01), respectively. Thus, even though we were unable to obtain information regarding adjuvant chemotherapy in our study, it appears that the use of chemotherapy cannot completely explain the survival differences observed in the histologic groups. Furthermore, the survival differences between these histologic cell types persist even in those with early stage disease where adjuvant chemotherapy is typically not employed. Another potential concern is that women with UPSC in our study received less adjuvant radiotherapy compared to CC and G3EC patients. Although this difference in radiotherapy may partially explain the poorer outcome of UPSC patients, we showed that radiotherapy did not impact the survival of our patients in multivariate analysis. Moreover, it is possible that many of the UPSC patients underwent chemotherapy rather than radiotherapy given that UPSC has a predilection for distant metastases (Cirisano *et al*, 1999; Huh *et al*, 2003; Hamilton *et al*, 2005). Despite the limited and incomplete information available on adjuvant treatment, our data reflect the lack of community standard which perpetuates inconsistency of approach and variation both within and between treatment centres.

The data from this population-based study allows one to generate interesting hypotheses. The strength in the large number of patients in this series may overcome potential limitations such as the lack of central pathology review and selection biases associated with large population-based analyses. Nevertheless, the demographic and clinico-pathologic data obtained from this report are a true reflection of the trends and outcomes of US women diagnosed with poor histologic uterine carcinoma who receive medical care from community hospitals based on diagnoses from contributing pathologists rather than from gynecologic pathologists in academic centres.

Our data suggests that UPSC and CC are histologically distinct tumours with aggressive tumour biology. Although current treatment modalities for these high-risk cell types have resulted in similar response rates compared to endometrioid tumours, UPSC and CC are discrete histologic subtypes that should be segregated from the more common endometrioid corpus cancers. In this manner, we can effectively design tailored therapies that may improve the outcomes of women diagnosed with these aggressive cancers.

## Figures and Tables

**Table 1:** Patient and treatment data

	**UPSC (*n*=1473)**	**CC (*n*=391)**	**G3EC (*n*=2316)**	***P*-value**
Median age (years)	70	68	66	*P*<0.0001
Race				
White	1152 (78%)	298 (76%)	1976 (85%)	*P*<0.0001
Black	213 (15%)	48 (12%)	164 (7%)	
Asian	77 (5%)	33 (8%)	132 (6%)	
Other	31 (2%)	12 (3%)	44 (2%)	
				
Stage^a^				*P*<0.0001
I	533 (36%)	197 (50%)	1388 (60%)	
II	171 (12%)	54 (14%)	252 (11%)	
III	268 (18%)	71 (18%)	353 (15%)	
IV	501 (34%)	69 (18%)	323 (14%)	

a Stage based on FIGO 1988.

UPSC=uterine papillary serous carcinoma.

CC=clear cell carcinoma.

G3EC=grade 3 endometrioid carcinoma.

**Table 2:** Multivariate analysis

**Factors**	**Hazard ratio**	**95% confidence interval**	***P*-value**
Stage of disease	2.05	1.93–2.17	*P*<0.001
Histology^a^	1.22	1.11–1.35	*P*<0.001
Age at diagnosis^b^	1.03	1.03–1.04	*P*<0.001
Race^c^	1.00	0.92–1.11	*P*=0.888
Adjuvant radiotherapy	0.99	0.93–1.03	*P*=0.450

a Uterine papillary serous carcinoma *vs* clear cell carcinoma *vs* grade 3 endometrioid carcinoma.

b As a continuous variable.

c Whites *vs* Blacks *vs* Asians.

**Table 3:** Studies comparing the survival of women diagnosed with high-risk corpus cancers

	**5-year survival rates**							
	**Year**	***n***	**Stage**	**UPSC (%)**	**CC (%)**	**UPSC+CC (%)**	**G3EC (%)**	***P*-value**
Carcangiu (Carcangiu and Chambers, 1995)	1995	76	I–II	40	68%			0.03
Cirisano (Cirisano *et al*, 2000)	2000	81	I–II	—	—	56	71	0.11
Alektiar (Alektiar *et al*, 2002)	2002	83	I–II	—	—	79	71	0.3
Halperin (Halperin *et al*, 2002)	2002	64	I–IV	62.5	—	—	80^b^	>0.05
Boruta (Boruta *et al*, 2004)	2004	96	I–IV	41^a^	—	—	75	<0.01
Creasman (Creasman *et al*, 2004)	2004	532	I	72	79	—	75	—
Hamilton	2005	2595	I–II	74	82		86	<0.0001
		1585	III–IV	33	40		53	<0.0001

a Greater than 50% UPSC.

b Includes grade 2 endometrioid carcinoma (*n*=19) and grade 3 endometrioid carcinoma (*n*=11).

UPSC=uterine papillary serous carcinoma.

CC=clear cell carcinoma.

G3EC=grade 3 endometrioid carcinoma.
